# Next-generation stress-inducible *Komagataella phaffii* promoter variants

**DOI:** 10.1186/s12934-025-02825-7

**Published:** 2025-10-30

**Authors:** Katharina Ebner, Núria Bernat-Camps, Simona Scheipel, Corina Dörner, Francisco Valero, Anton Glieder, Xavier Garcia-Ortega

**Affiliations:** 1 Bisy GmbH, Wuenschendorf 292, Hofstaetten/Raab, 8200 Austria; 2 Department of Chemical, Biological and Environmental Engineering, Universitat Autònoma de Barcelona, Bellaterra (Cerdanyola del Vallès), 08193 Spain; 3 Austrian Centre of Industrial Biotechnology (ACIB), Graz, Austria; 4 Institute of Molecular Biotechnology, Graz University of Technology, NAWI Graz, Petersgasse 14, Graz, 8010 Austria

**Keywords:** *Komagataella phaffii* (*Pichia pastoris*), Promoter engineering, Recombinant protein production, Methanol-free expression system, Stress response promoter, *HSP12* promoter, Block-scanning technologies

## Abstract

**Background:**

Expanding the promoter toolbox of *Komagataella phaffii (K. phaffii)* in terms of strength and regulatory flexibility can significantly enhance bioprocess efficiency for recombinant protein and metabolite production. The most frequently used promoters are still derived from the methanol utilization (MUT) pathway or genes of the central metabolism. However, the hazards and costs associated with methanol have prompted the search for alternative promoters, including engineered variants. A key limitation remains, many available promoters are still growth-coupled, tying production to biomass accumulation and shortening process duration. Promoters with growth-decoupled expression are therefore highly desirable. In this context, the recently described P_DH_ promoter is of interest due to its methanol independence, strong expression, and growth-decoupled regulation.

**Results:**

In order to identify potential activator sites of the P_DH_, a systematic semi-rational block-scanning approach was used, employing single-base and sequence block walking mutagenesis. The strength of 152 systematically generated variants was characterized using the intracellular reporter eGFP. Variants showed altered strengths and regulatory patterns with fluorescence levels spanning approximately 10–150% of the parental promoter. Subsequently, the best-performing variants were combined to multi-combination variants, which showed activities up to 250% of the parental P_DH_. Selected variants were also evaluated with the industrially relevant and secreted enzyme *Cal*B, a lipase from *Candida antarctica.* Lipase product titers were approx. 2-fold higher than with the parental native promoter sequence and also outperformed the typical state-of-the-art benchmark and constitutive and growth-coupled *GAP* promoter (P_*GAP*_).

**Conclusions:**

Creating and characterizing variants of the P_DH_ sequence supported the elucidation of the sequence-function relationships of this promoter. In addition, the surprisingly beneficial effects of a synthetic 10 bp sequence stretch opened up opportunities for further engineering of this system and extended the toolbox of efficient vector parts for methanol-free and growth-decoupled protein production with *K. phaffii*. Those additional promoter sequences will also support the construction of stable engineered strains with a balanced expression of multiple genes, as needed for e.g. multienzyme pathways and synthetic biology applications.

**Supplementary Information:**

The online version contains supplementary material available at 10.1186/s12934-025-02825-7.

## Background

*Komagataella phaffii* (*K. phaffii*, previously *Pichia pastoris*) is best known for the efficient production of clean secreted proteins [1]. Additionally, typical eukaryotic posttranslational modifications such as N-glycosylation and disulfide bond formation are enabled. Today, this methylotrophic yeast is amongst one of the most relevant microbial hosts for recombinant protein production (RPP), rivaling *E. coli* and surpassing *S. cerevisiae* for some target groups [1, 2]. Over the years, its value as an industrial expression host was proven multiple times: from the manufacturing of the catalytic subunit of bovine enterokinase (EK_L_) [3] and hydroxynitrile lyase (HNL) [4] (> 20 g/L) in the early 90s as the first large-scale RPP processes, up to the more recent production of challenging targets such as biopolymers, dairy proteins and biologics e.g. collagen [5] human and bovine lactoferrin [6], antimicrobial peptides and cytokines [7]. Since the COVID-19 pandemic, *K. phaffii* is also used for quick and simple production of recombinant SARS-CoV-2 receptor binding domain and variants thereof, applied as vaccine candidates [8–11]. Up to now, more than 600 proteins of different origins (viruses, bacteria, fungi, plants, invertebrates, vertebrates) have been made by *K. phaffii*, many of them with relevance in the food, feed, and pharmaceutical industry [12–14].

In the last two decades, many academic research groups and companies have contributed to advancing this expression system by engineering various parts, including expression chassis/strains, tools and methods for genomic manipulation, and regulatory sequences — some of which are summarized in recently published reviews [15–17]. Various different promoters have successfully been employed for heterologous protein production with *K. phaffii*. Most frequently used state-of-the-art promoters were derived from the endogenous methanol utilization (MUT) pathway [18], housekeeping genes (P_*ENO1*_ [19]), central metabolism (P_*GAP*_ [16]) or other highly transcribed genes as identified from transcriptome analyses (P_*GCW14*_/commercial P_*UPP*_ [20, 21], P_*GTH1*_ [22]) and are either constitutive, derepressible or methanol-induced. Also, orthologous promoters from other yeasts [23] and synthetic variants [24–26] contributed significantly to the promoter toolbox for strong expression. Two frequently used promoter sequences for this expression system are the methanol-inducible P_*AOX1*_ (alcohol oxidase 1) and the constitutive P_*GAP*_ (glyceraldehyde-3-phosphate dehydrogenase promoter) [16], which are also commonly used as benchmarks for comparative studies employing new methanol-inducible and methanol-independent promoters, respectively. The P_*AOX1*_ is strong, tightly regulated and, without a doubt, the best-studied promoter sequence of *K. phaffii*. It has successfully been used for the recombinant production of a multitude of different proteins in all applicable scales – from microscale to commercial production plants. Nevertheless, using wildtype P_*AOX1*_ drawbacks can arise due to the need for methanol as an inducer of high-level expression, which is toxic and flammable and can pose safety challenges at high concentrations and large quantities [15, 16]. In contrast, the P_*GAP*_ with its glycolytic nature does not need hazardous inducers and leads to a strong constitutive expression, coupled to growth, with the carbon sources glucose or glycerol. However, this strict growth-coupled protein production causes challenges with respect to efficient energy management and carbon efficiency, and is problematic for the expression of cytotoxic proteins [27, 28]. Although the P_*AOX1*_ and P_*GAP*_ are complementary in many properties and constraints, a broader set of regulatory elements is needed to precisely fine-tune promoter-target gene combination, which is a key concept to increase recombinant protein yields [29–33]. In the last two decades, many studies have contributed to this expanded toolbox of promoter elements for *K. phaffii*, reporting novel natural elements as well as engineered variants.

For *de novo* discovery, microarray analysis as well as transcriptome and genome sequencing were used to identify novel promoters with different regulatory profiles, including constitutive [29], P_*PDC*_ and P_*PYK*_ [30]) and novel inducible sequences (P_*CTA1*_/commercial P_DC_ [31–33], P_*LRA3*_ [34], P_*MOX*_ [35], P_*HSP12*_/commercial P_DH_ [36]).

Besides *de novo* discovery, also the engineering of established promoter sequences was explored to expand the promoter toolbox of *K. phaffii*, as it allows the retention of pre-existing characteristics and is usually less time and cost-intensive. Generally, eukaryotic promoter engineering is mostly focused on the modification of already existing sequences or the utilization of native scaffolds. Ab initio design or design from scratch is uncommon due to the common incomplete understanding of regulatory circuits and sequence-function relationships [37, 38]. Despite the high uncertainty if mutations can change promoter strength or regulatory properties, deletions and exchanges of short DNA stretches, as well as truncations proved to provide relatively simple routes to improve the performance of native promoters. The fundamental concept of all commonly employed strategies (rational, random or combination) is to influence transcription by deletion, insertion or duplication of transcription factor binding sites (TFBS) of a promoter sequence or changing the context of their occurrence [39]. However, based on the analysis of expression also alternative effects can be potential reasons for changes in target protein yield, like differences in mRNA stability or translational initiation [37, 40]. 

An early attempt to systematically expand the repertoire of *K. phaffii* promoter sequences by engineering was performed by Hartner et al. with synthetic mutations and deletions of the P_*AOX1*_ [24]. Generally, due to its large popularity, strength and tight regulation, the P_*AOX1*_ is a prime example for promoter engineering efforts. A multitude of different engineering approaches were applied, including but not limited to random mutagenesis [41], deletion variants [42], hybrid and partially synthetic hybrid promoters [40, 43] as well as manipulation of putative TFBS/cis-acting elements [24]. All of these approaches resulted in libraries of various sizes and expression strengths. Rational approaches, for example, reported by Hartner et al. (2008), Vogl et al. (2014), and Portela et al. (2018), resulted in libraries spanning approximately 6-160%, 10-117% and 20-140% of the parental promoter under methanol induction, respectively [24, 40, 44].

Promoter engineering trials not only result in novel regulatory sequences, but they can also help elucidate function-structure relationships and thereby identify targets for further development, including cis- and trans-acting elements as well as whole regulatory circuits [24–26, 45]. Successful examples for *K. phaffii* include the various methanol-free P_*AOX1*_-based systems reported in the last decade, as well as a study done by Özge et al. (2017) resulting in a 2-fold increased expression from a P_*GAP*_-based system by overexpression of a newly identified TF [25, 31, 46, 47].

In summary, many successful efforts have been made to engineer the classical methanol-inducible *AOX1* and constitutive *GAP* promoter of *K. phaffii*. In contrast, reports about engineering campaigns focused on alternative *K. phaffii* regulatory sequences are scarce. Notable exceptions are the engineering of the P_*ADH*_ [48, 49] and a small library of P_*CTA1*_ variants [26]. In this work, the aim was to engineer the P_DH_ promoter sequence, which had been described by Bernat-Camps et al. [36], to investigate the sequence-function relationship of this growth-decoupled, methanol-independent sequence and benchmark it against current state-of-the-art methanol-independent promoter systems [36].

## Results and discussion

### Systematic scanning of the P_DH_ results in novel promoter variants with up to 150% of activity

Recently, discovery of the novel P_DH_ promoter system for recombinant protein production (RPP) with *K. phaffii* was described. It consists of a 352 bp upstream region of the endogenous heat shock protein 12 (*HSP12*) gene. This region was identified as the shortest functional promoter capable of driving high expression levels, particularly notable under severe carbon source-limited conditions due to elevated transcript levels of *HSP12*. Employing this promoter for recombinant *Ca*lB production revealed an inducible and non-growth coupled expression profile, which outperformed the methanol-independent P_*GAP*_-based reference 2.3-fold with respect to the lipase titer [36]. Additionally, literature reports underline its involvement in stress responses by the observed alkaline-induced *HSP12* expression in baker’s yeast [50]. Taking together its strength, favorable regulatory pattern and small size, the P_DH_ was considered a promising candidate to evaluate different engineering approaches to create novel sequence-diversified promoter variants, as well as identify neutral regions and regulatory hotspots, and potentially improve promoter strength.

**Fig. 1:**
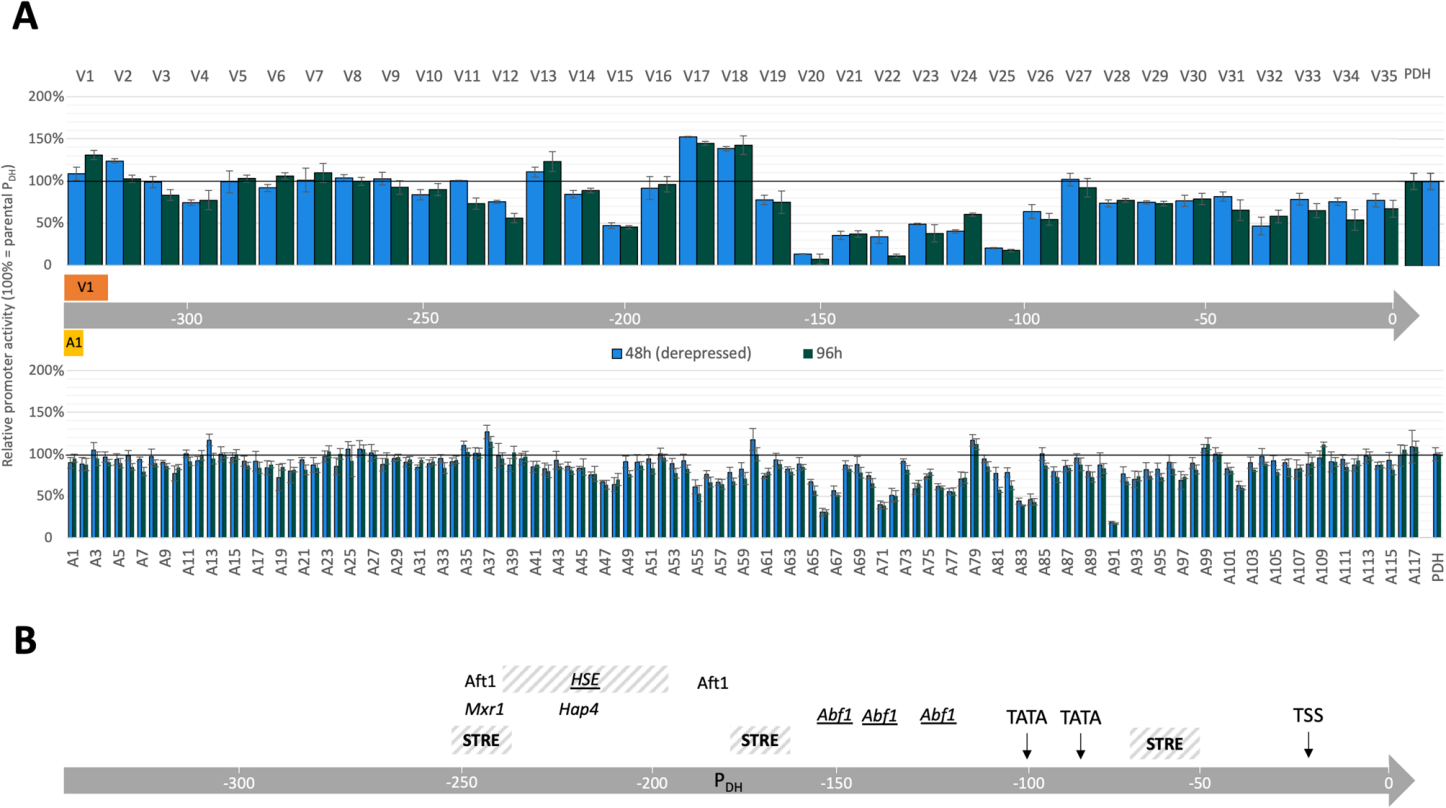
**A** Semi-rational systematic scanning of the P_DH_ with small sequence replacements using two different sequence blocks, V (5′ GATAACCGTG 3′) and A (5′ AAA 3′). Initial evaluation of all variants was done with intracellular eGFP as reporter in high-throughput microscale 96-well plate assays. Relative promoter activity is shown as OD_600_ corrected fluorescence normalized to the parental P_DH_ (RFU OD_600_^-1^ PDH^-1^) output upon full carbon source depletion (48 h) and after further 48 h of cultivation with low carbon source pulse-based feeding. Given values represent the mean of biological triplicate cultivations of a representative strain, standard deviations are shown. **B** Schematic representation of putative regulatory sites. Putative TATA boxes, TATA box-like sequences, TFBS predicted by manual sequence alignments, as well as the transcription start site (TSS) are indicated. Putative TFBSs and cis-acting elements predicted by PROMO (italic, underlined), JASPAR (bold) and YEASTRACT (italic) are annotated

In a preliminary study using the *K. phaffii* promoter P_*CTA1*_ (also P_*CAT1*_) [18], two different random 10 bp sequences, V (5′ GATAACCGTG 3′) and Z (5′ ATCCTTTTAG 3′), were evaluated as sequence blocks for a systematic block-scanning approach spanning the whole promoter with sequence replacements. Surprisingly, the V-sequence block showed superior results compared to the Z-sequence block, as 34% of all V-variants showed higher promoter activity compared to the corresponding Z-variants (unpublished data), marking the V-sequence as an attractive engineering tool for further work. Subsequently, for the engineering of the P_DH_, a semi-rational systematic approach was applied, where the whole sequence was scanned with small replacements using two sequence blocks of different lengths. The first was the 10 bp long V-sequence block (5′ GATAACCGTG 3′) and the second was a 3 bp long repetition of Adenines (5′ AAA 3′), similar to the approach described by Portela et al. [44]. Using the V-sequence block, a library of 35 variants (V-library) was generated by systematically exchanging every ten bases of the 352 bp long P_DH_ against this sequence. The resulting variants were named V1-V35 with the numbering starting at the 5′ end of the promoter. The same procedure was followed with the A-sequence block, creating 117 variants (A-library) by sequentially exchanging every three bases of the P_DH_ with three Adenines, giving rise to variants A1-A117 and applying the same numbering rule. An initial evaluation of all variants was done using intracellular eGFP, a widespread reporter system for which a direct correlation between fluorescence and transcript levels has been reported previously [51]. To reliably compare regulatory elements, we aimed for targeted single copy genomic integration of the expression cassettes into the *AOX1* locus using a *KU70* deficient *K. phaffii* strain and 1000 bp long homologous sequences on the expression vector [52]. The clone screening was performed in a high-throughput (HT) microscale system in a methanol-free cultivation setup. Fluorescence levels, normalized by cell density, were determined after carbon source depletion (48 h) and after a further cultivation period of 48 h with low carbon source pulse-based feeding. To facilitate the comparison of variants against each other and to the parental promoter regarding eGFP production, relative promoter activity, normalized to the parental P_DH_-GFP strain fluorescence (RFU OD_600_^−1^ P_DH_^−1^) was calculated for each timepoint (Fig. 1A).

**Fig. 2:**
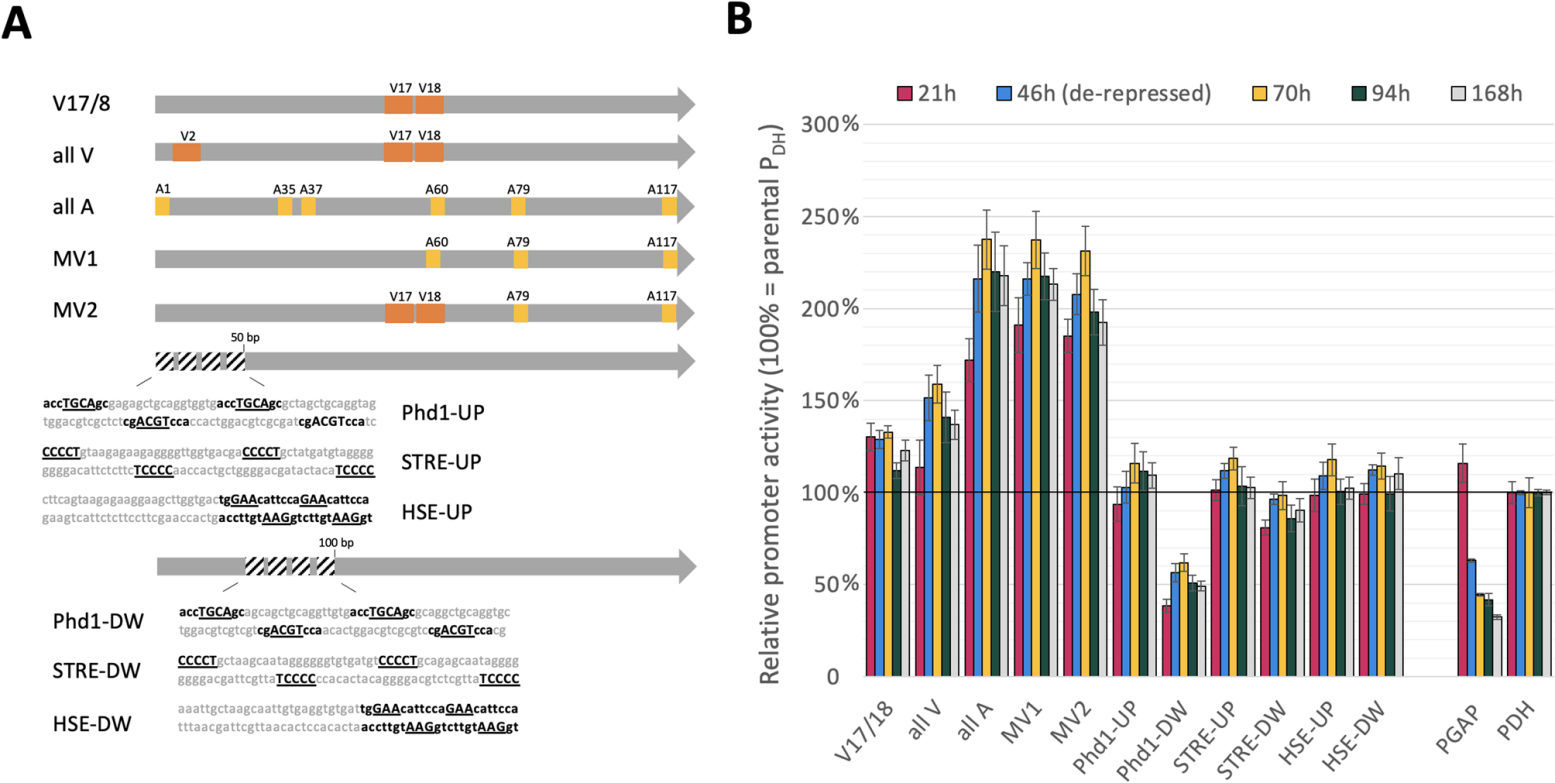
Second generation P_DH_ variants, schematic representation **(A)** and initial determination of promoter activity in high-throughput microscale using intracellular eGFP as reporter **(B)**. **A** Eleven second generation variants were created, five multicombination variants (combination of interesting V- and A-variants) and six variants with additional cis-acting elements as replacement of the wildtype sequence. The cis-acting elements HSE (`CCCCT´), STRE (`GAA´) and the Phd1 binding site motif (`TGCA´) were positioned in four consecutive repeats alternating between the two DNA strands at two different locations in the most upstream part of the P_DH_ (UP = 1-50 bp or DW = 50-100 bp). **B** Initial evaluation was again done with intracellular eGFP in high-throughput microscale 96-well plate assays. Relative promoter activity is shown as OD_600_ corrected fluorescence normalized to the parental PDH (RFU OD_600_^-1^ PDH^-1^) and was determined at five different points of the cultivation, starting as soon as 21 h and ending after 168 h. Given values represent the mean of biological triplicate cultivations of a representative strain, standard deviations are shown.

Variants of both libraries showed altered strengths and regulation patterns compared to the parental promoter with overall activities spanning from 10-150% of the parental P_DH_. Generally, triple adenosine exchanges were less disruptive for the sequence-function relationship of the promoter than 10 bp exchanges. This could be related to the low GC content of the wildtype P_DH_ (GC ~ 37%), as typical for promoter sequences of lower eukaryotes and high-activity promoters [53]. Case in point, for the A-library only around one-third of all variants (34 at de-repression, 40 after feeding) have distinctively altered promoter strengths, while all others varied between 80% and 120% of the parental sequence. In contrast, for the V-library the opposite had been observed. More than half of the variants (21 at de-repressed and 24 after feeding) present activities lower or higher than 80% or 120% compared to the parental P_DH_ benchmark, respectively.

The highest activities were determined for two variants of the V-library, V17 and V18, which changed adjacent regions of the P_DH_. Interestingly, only one of the six corresponding variants from the A-library showed increased expression, whereas the remaining five A-variants presented activities lower than 80% of the parental promoter. Furthermore, for most variants both sampling points (de-repression, feeding) show the same relation to the parental P_DH_, indicating a similar regulatory pattern. Only a few variants show pronounced differences for the two time points (de-repression, feeding) suggesting de-regulation of the promoter (V1, V3, V2, V11, V12 and A13). However, none of them have corresponding variants in the second library with similar expression profiles. 

Such distinct sequence block-specific effects–i.e., variants from the two libraries having modifications in the same region showing vastly different expression results–were observed several times throughout both libraries. Therefore, we assumed that the observed expression effect was not solely caused by an interruption of potential activator or repressor binding sites, but by a potential positive effect of the inserted V-block. 

Although chosen as a random sequence block, further analysis of the 10 bp V-sequence revealed some similarity to the 5’ end of a potential binding site (GATA[A/T][A/G/T]) for Gln3p, a transcriptional activator of *S. cerevisiae* with a predicted homolog in *K. phaffii*. However, not all V-variants show enhanced expression, which could be due to the fact that there is no perfect match for the whole consensus, the specific binding site of *K. phaffii* Gln3p is not known and/or regional and spatial TFBS positioning has to generate a fit with the overall promoter architecture.

Furthermore, effects related to exchange block size and compositions as well as the introduction of novel TFBS have to be considered. Regarding block size, 10-12 bp have previously been described as a critical length for promoter sequences since they represent approximately one helical turn of DNA. This should reduce effects related to the three-dimensional orientation of cis-acting elements and/or the interaction of different trans-acting elements with each other [54]. 

Lastly, on a more holistic level, sequence changes can lead to alterations in DNA packaging by the way of influencing nucleosome positioning. The addition of poly(dA: dT) tracts in the A-library and potential removal of them in the V-library might affect the nucleosome occupancy throughout the whole promoter sequence, similar as previously reported by Yang et al. as an engineering strategy for the *K. phaffii* P_*AOX1*_ [55].

### Regulatory hotspots of the P_DH_ and the implications for transcriptional control

Besides the discussed sequence block-specific effects, both libraries highlighted approximately the same regions for positive, negative or neutral influence of sequence modifications. To better understand the sequence-function relationship of these regions and therefore acquire a more comprehensive understanding of the cis- and trans-acting elements potentially involved in P_DH_ regulation, the whole sequence (with special regard to influenced regions) was analyzed in silico for putative TFBS using PROMO, JASPAR and YEASTRACT (Fig. 1B). With those tools, the search was focused on cis- and trans-acting elements of fungi or *S. cerevisiae*, since database information on these elements from *K. phaffii* is limited (only one entry in YEASTRACT). The analysis was centered on cis- and trans-acting elements involved in carbon source-related stress conditions since the cultivation conditions applied in this study were focused on promoter activation by carbon restriction. Potential regulatory elements for alternative activating/repressing conditions of *HSP12* (e.g. salt, pH, temperature) are not discussed in detail.

Briefly, in the first 100 bp of the promoter (-352 to -252 from the CDS) corresponding to variants V1-10 and A1-35, 90% of the sequence exchanges did not result in drastic changes in promoter activity. This suggests no larger stretches of major cis-acting elements are involved in the regulation of basal activity.

Sequence exchanges in the following 90 bp (-252 to -162 from the CDS), represented in variants V11-V19 and A37-A64, resulted in a strong but not consistently good or bad influence on promoter strength, highlighting this region as a potential regulatory hotspot. Analysis of the P_DH_ for potential TFBS revealed potential binding sites for stress and carbon source response elements in this region, as well as for factors involved in preinitiation complex (PIC) assembly, a complex necessary for initiating gene transcription. The consensus binding site (´VGGGG´) for the general stress response TF Msn2p, also known as stress-responsive element (STRE), was found two times within this region (V11/12, V19) and once further downstream (V29/30). For all three putative STREs within the P_DH_, corresponding variants with promoter activities below 80% of the parental sequence were observed, suggesting that this motif plays a role in PDH regulation. For the most upstream STRE (V11/12) only one encompassing V-library variant (V12) shows overall decreased activity compared to the parental sequence, but both V11/12 and an A-library variant (A37) show lower promoter activity after carbon source feeding (96 h) than after initial carbon source depletion (48 h), indicating altered promoter regulation. Interestingly, this region (V11-V12) carries a putative binding site for the *K. phaffi *carbon source responsive TF Mxr1p (methanol expression regulator 1) (-245 to -239 from the CDS), also called *Kp*Adr1, a homolog of *Sc*Adr1p. *Kp*Mxr1p is one of the best-studied TF factors of *K. phaffi* and a global regulator involved in the control of various metabolic pathways, most prominently the MUT pathway, but also acetate metabolism, peroxisome biogenesis and fatty acid oxidation [56–58]. Furthermore, also for the region represented by variants V14-V15 (-222 to -202 from the CDS) multiple putative TFBS could be identified in silico. The region contains the consensus sequence elements related to PIC assembly, however, with the translation start site more than 200 bp away and no TATA-box-like motifs, it seems unlikely that this region is the major site for PIC assembly for the *HSP12* gene. Furthermore, YEASTRACT revealed a putative binding site for *Sc*Hap4p in this region, which is a transcriptional activator and global regulator of respiratory genes in response to carbon source. Also, broadening the search parameters in PROMO to allow for the annotation of all eukaryotic TFBS, a putative Hsf1p binding site was revealed, which is known as heat shock element (HSE). Upon closer examination of the sequence, various repeats of the HSE core motif ´GAA´ can be found on the leading and lagging DNA strand in the area 123-161 bp represented by variants V13-V17 (-230 to -192 from the CDS). Although HSEs are generally known as highly ordered multiple repeats of the core motif arranged in alternating orientations with consistent spacing (nnGAAnnTTCnnGAAnn), also a multitude of “noncanonical” HSEs have been described for yeast promoters previously [59]. Since heat shock transcription factors (HSF) are described to be permanently bound to HSEs even in a non-active state [60], the varying effects observed for variants V13-V17 (-230 to -192 from the CDS) could be an indication for the occupation of HSEs by HSFs causing or resolving steric hindrance for the transcription machinery. Finally, based on the activity of variant V18 (-180 to -174 from the CDS) only one TF potentially involved in the negative regulation of the P_DH_ could be identified in this "regulatory hotspot" region. This TF is Aft1/2p, which in *S. cerevisiae* is described as a transcriptional activator involved in iron-dependent regulation of genes with the recognition motif ´RCACCC´ [61].

Finally, except for isolated variants, all modifications in the last 160 bp of the promoter (-162 to -1 from the CDS), represented in variants V20-V35 and A65-A117, consistently showed a negative influence on promoter activity, which was especially pronounced for V20-V25 (-162 to -102 from the CDS). Here, some variants only showed a remaining activity of 10% compared to the parental P_DH_, which indicates the importance of this region for transcription. No TATA-boxes or similar motifs [62] could be found in this region, only three putative *Sc*Abf1p binding sites were indicated by in silico analysis. For *S. cerevisiae HSP12* one putative Abf1p binding site was described, however, subsequent mutation studies indicated no significant influence of this TFBS on *HSP12* transcription [63]. Analysis of the region V26-V28 revealed two potential sites for initiation of PIC assembly, one fitting the yeast TATA-box consensus sequence ´TATAWAWR´ and the other showing a TATA-like motif. For both motifs, V- and A-variants with activities decreased by at least 30% can be found (V26/A84 and V28/A91). This effect is especially pronounced for A91, which carries changes in the most conserved part of the putative TATA-box (´TATAWAWR´ to ´AAAAWAWR´). Additionally, for both motifs the position fits with the typical yeast promoter architecture: TATA-box(es) 80-100 bp upstream of the translation start site and 50-70 bp away from a potential TSS (-30 to -20 from the CDS) [39]. Lastly, also the low promoter activity of the successive variants V28-V33 (-82 to -22 from the CDS) supports this presumption, since a high sensitivity of the whole functional region involved in PIC assembly has been described previously [40]. 

Shortly summarized, this work allowed to identify one major positive cis-acting element in the P_DH_ at the region 190-250 bp (V20-V25, -162 to -102 from the CDS) as well as further minor positive cis-acting elements in the surrounding areas: 100-120 bp (V11-V12,-252 to -232 from the CDS), 130-150 bp (V14-V15, -222 to -202 from the CDS) and 280-300 bp (V29-V30, -72 to -52 from the CDS) under carbon source limitation. It is important to highlight that their identification might be the result of the combination of several effects, which include altering or adding cis-acting elements and/or affecting their three-dimensional displacement, as well as changing accessibility by nucleosome repositioning or other minor interactions. Also, it is essential to bear in mind that with 352 bp, the P_DH_ is a rather short regulatory sequence, additional cis-acting elements could also be located further upstream of the gene. As a case in point, a region sensitive to alkalinization was recently identified between 1000 bp and 450 bp upstream of the *HSP12* CDS [64]. Furthermore, three potential sites for initiation of PIC assembly could be identified, whereby two of them contain a TATA-box or TATA-box-like motif, and are 80-100 bp upstream from the CDS, fitting with the general yeast promoter architecture. In silico analysis of the promoter revealed consensus sequences for binding sites of Abf1p, Msn2p, Mxr1p, Hsf1 and the Hap complex – all potentially involved positive trans-acting factors – as well as Aft1p – a potential negative trans-acting factor. However, additional experimental studies will be needed to verify these predictions. In *S. cerevisiae*, *HSP12* expression upon salt and heat shock stress is regulated by the Hog (high-osmolarity glycerol) and Ras/PKA pathway, with Msn2/4p identified as major TF governing activity by binding to STREs. However, under glucose de-repressing conditions there is an unidentified TF binding these regulatory elements. No major involvement of HSEs and therefore Hsf1p could be found under these cultivation conditions. However, even in cells lacking all four activators of the Hog pathway (Msn2p, Msn4p, Hot1p and Sko1p), expression from the promoter could be induced, which indicates a complex and so far, not yet completely unraveled activating system [65].

### Combination of beneficial mutations results in second-generation P_DH_ variants with two-fold increased expression

After scanning the whole P_DH_ with small sequence exchanges, creating variants with altered strengths and identifying regions suitable for modifications, there was an interest to evaluate if the gained understanding and knowledge can be used to engineer new promoter variants to enhance the expression of typical commercially used proteins. Therefore, two strategies were pursued, (1) a combination of V- and A-library variants with interesting and/or enhanced expression pattern creating multicombination variants to evaluate potential additive effects and (2) the integration of novel cis-acting elements. In total, eleven novel variants (multi-variants) were created this way, five multicombination variants, and six variants with additional cis-acting elements (by replacement of wildtype sequence to minimize structural effects) (Fig. 2).

**Fig. 3:**
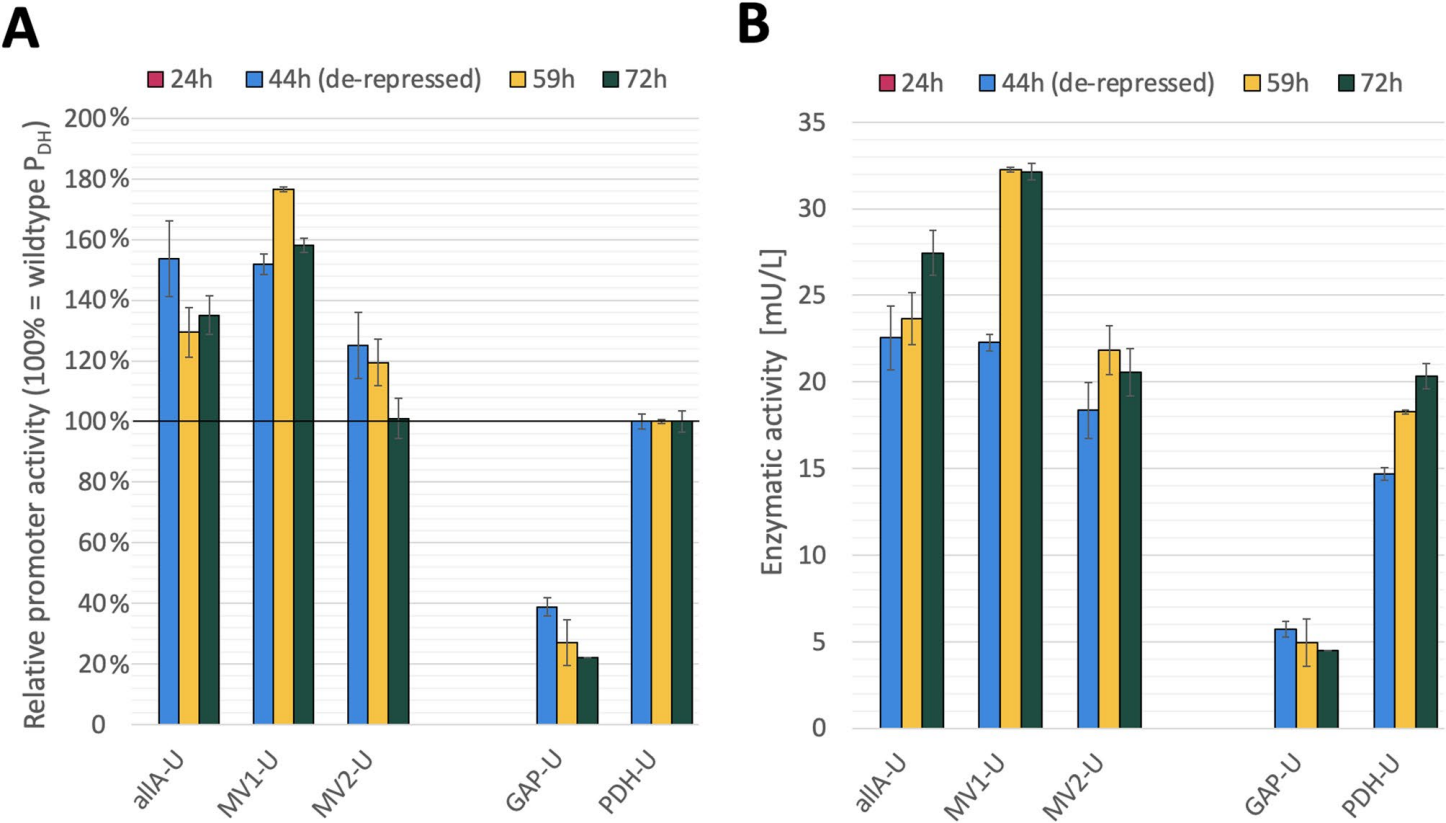
Evaluation of selected second generation P_DH_ variants in shake flask scale with the secreted model *Abr*UPO. **A** Relative promoter activity, represented by mean enzymatic activity normalized to the value of the PDH-U (U mL^-1^ P_DH_^-1^). **B** Enzymatic activity in the supernatant, shown as volumetric activity (mU/L^-1^). Enzymatic activity of three multivariants (AllA-U, MV1-U and MV2-U) is compared to the benchmarks GAP-U and PDH-U. Shake flask cultivations were conducted in triplicates under severe carbon-limiting conditions (pseudo-starving), which were applied after 24 h of batch phase by the addition of a single glycerol FeedBead^®^. The error bars represent standard deviation of biological triplicate cultivations.

Briefly, as cis-acting elements, the HSE (`GAA´), the STRE (`CCCCT´) and the Phd1 binding site motif (`TGCA´) were chosen and positioned at two different locations in the most upstream part of the P_DH_ (1-50 bp or 50-100 bp) in four consecutive repeats alternating between the two DNA strands (Fig. 2A). Regions 1-50 bp (UP) and 50-100 bp (DW) were chosen for the addition of the cis-acting elements due to the minimal effects observed upon sequence exchanges during the promoter scanning approach. The novel elements were placed in tetramers to imitate the multiple copies of putative P_DH_ regulating cis-elements (e.g. Abf1p, STRE) revealed by sequence analysis. Initial evaluation of these second-generation variants was again performed with intracellular eGFP in microscale under de-repressed conditions, as described previously. Samples for fluorescence measurements were taken over a cultivation period of 168 h, starting as soon as 21 h.

For the parental P_DH_ and most variants a steady increase in eGFP amount could be seen over the whole cultivation time, which emphasizes the drawn-out protein production window of these promoter sequences (Supplementary File 1: Figure S 1). In contrast, for the constitutive benchmark, P_*GAP*_, fluorescence levels stay constant over time revealing a rather short protein production window at the very beginning of the cultivation, in agreement with its growth-coupled regulation.

Furthermore, only the multicombination variants showed enhanced promoter activity, while variants with additional cis-acting elements showed eGFP expression comparable with or lower than the parental promoter (Fig. 2B). No benefit was recognized by the additional implementation of known activator binding sites. The multicombination variants allA, MV1 and MV2, showed the highest overall values with promoter activities ranging from 200-225% compared to the P_DH_ (at different timepoints) and two- to six-fold higher expression than the constitutive benchmark P_*GAP*_ (Fig. 2B). Interestingly, the multicombination variant P_DHV17/18_ carrying the two best performing single-exchanges, V17 and V18, showed a lower activity (110-130% of the P_DH_) than the individual single-exchange variants. There is no immediately palpable reason for this, however, double repetition of the 10 bp exchange sequence in close proximity could impact overall sequence integrity or interfere with DNA conformation. In contrast, for the combination of A-library exchanges, there was a clear synergistic effect. The best multicombination variants all contain the A-library exchanges A79 and A117, which as individual variants only showed slightly increased promoter activity (110-120% of the parental P_DH_).

After initial characterization of the second-generation variants with intracellular eGFP, the transferability of the obtained results for alternative protein products was evaluated. Here, as an example for a secreted protein, we chose *Abr*UPO (OJJ73116.1 [66]), an unspecific peroxygenase that can easily be detected by a colorimetric HT assay (oxidation of ABTS) and has previously been produced with *K. phaffii* to a titer of almost 1 g L^−1^ in fed-batch cultivation [67]. Initial screening of all eleven second-generation P_DH_ variants in microscale and comparison to the parental P_DH_ revealed the same trend as observed with eGFP, but an overall lower improvement with the variants (Supplementary File 1: Figure S 2). It is important to bear in mind, that a direct correlation between transcript amount and protein yield is more likely to be seen for intracellular proteins. Proteins that require complex and demanding folding and/or are secreted, often only show a linear correlation until limitations for membrane translocation, signal sequence processing and folding within the ER are met [68]. For secretory expression, similar as for copy number, optimal rather than maximal promoter strength is desired to balance transcript amount and reach maximal yield.

For the three most promising variants, an average clone was chosen to evaluate upscaling performance, furthermore referred to as strains allA-U, MV1-U and MV2-U. Previously, Vogl et al. showed that for *K. phaffii* screening landscapes averagely performing clones can be seen as representative due to the low percentage of outlier clones caused by specific integration events [68]. Cultivation of allA-U, MV1-U and MV2-U was scaled up to shake flasks employing the severe carbon-limiting protocol established for the parental P_DH_ to evaluate promoter performance under production conditions [36]. Here, allA-U and MV1-U showed a pronounced improvement of approximately 40-60% and 60-80% at different sample points, respectively, compared to the parental P_DH_-based *Abr*UPO-producing strain (PDH-U) (Fig. 3A). This relates to a four- to seven-fold higher enzyme titer than for the methanol-independent *Abr*UPO-producing strain employing the P_*GAP*_, GAP-U.

**Fig. 4:**
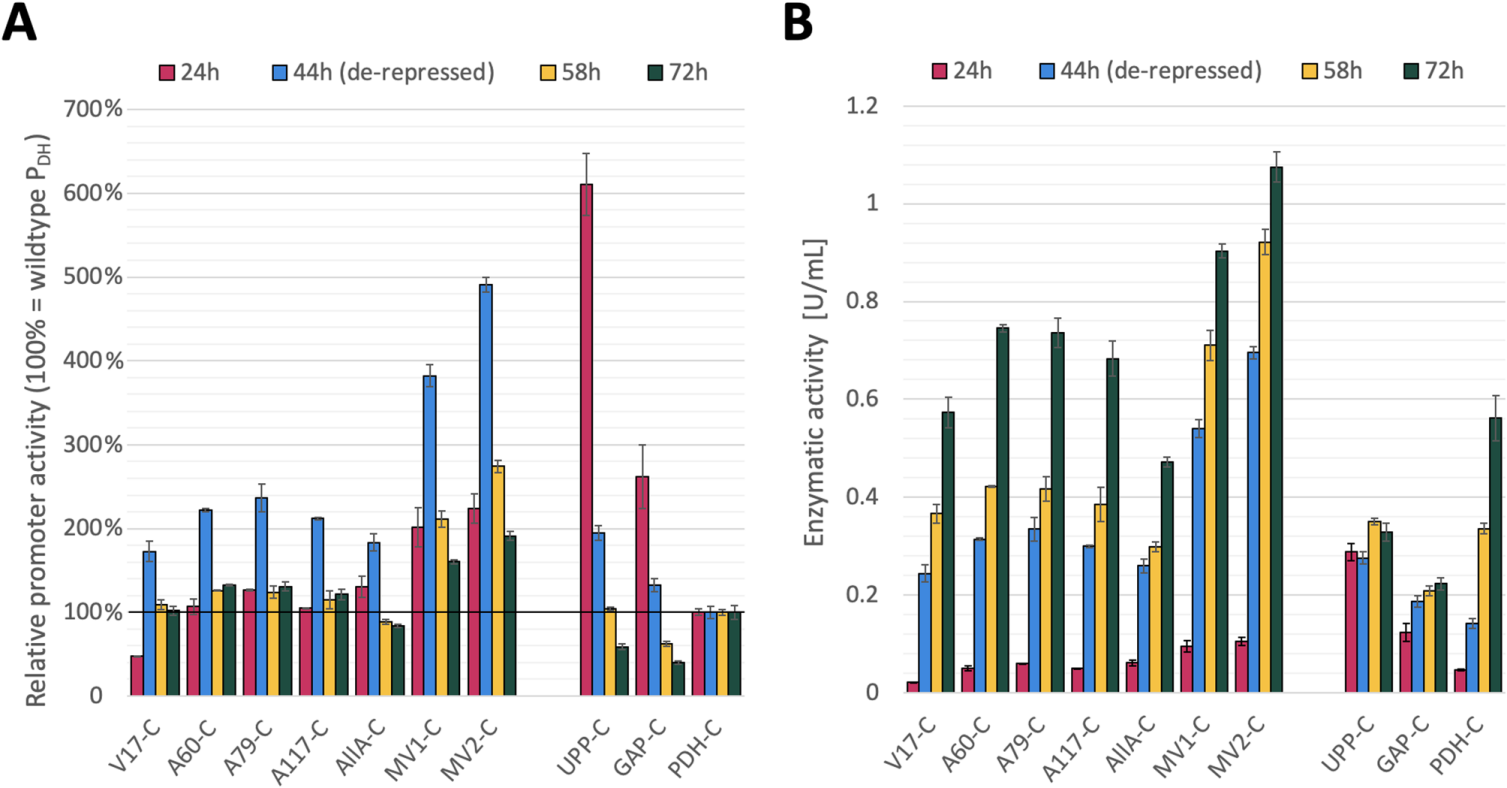
Evaluation of *Ca*lB production by selected P_DH_ variants in shake flask cultivations in terms of enzymatic activity. **A** Relative promoter activity is shown as mean enzymatic activity in the supernatant normalized to value of the PDH-C (U mL^-1^ P_DH_^-1^). **B** Lipolytic activity in the supernatant shown as volumetric activity (U mL^-1^). The performance under pseudo-starving conditions of four single variants and three multi-variants was assessed and compared to the benchmarks UPP-C, GAP-C and PDH-C, whereby the first two are growth-coupled systems and the latter is growth-decoupled. After the 24 h. of batch phase, one glycerol FeedBead^®^ was added to each shake flask to reach pseudo-starving conditions. The error bars represent standard deviation of biological triplicate cultivations

In contrast to the intracellular model eGFP, for *Abr*UPO, no reporter enzyme activity was detectable after 24 h of cultivation (batch phase), not even for the growth-coupled GAP-U. After 20 h of de-repression (sample point 44 h), MV2-U showed a 20% higher peroxidase activity than PDH-U, whereas allA-U and MV1-U display 50% higher values. Interestingly, while the activity of allA-U and PDH-U continuously increased over time, MV1-U and MV2-U reached their maximum at 59 h (Fig. 3B). The final total protein concentration in all cultivations was in the 13-23 mg L^−1^ range, which is similar to the titer achieved with different UPOs in methanol-induced shake flask cultivations [69]. Overall, for two of the three transferred second-generation variants (represented by strains allA-U and MV1-U), results obtained with the secreted model *Abr*UPO showed the same positive trend as with intracellular eGFP although with a lower total increase. For the third variant, represented by strain MV2-U, the performance did not resemble the trend described with eGFP. Here, we speculate that the special growth conditions (microscale vs. shake flask), differences in processing of the two models (intracellular vs. secreted) or mRNA stability – as a result of promoter-reporter-terminator sequence combinations – might be additional factors influencing production performance.

### Production of recombinant CalB achieved with P_DH_ variants outperformed classical methanol-free systems two-fold

Finally, the most promising P_DH_ variants were used to recombinantly produce lipase B of *Candida antarctica* (*Ca*lB), one of the industrially most relevant lipases used in various biocatalytic procedures. To facilitate a more accurate comparison of these new variants with previous promoter studies utilizing the *Ca*lB as a reporter protein, the same background strain and clonal strategy were used. Therefore, isogenic strains controlling *Ca*lB expression by the seven most interesting P_DH_ variants were generated, as reported previously for PDH-C, GAP-C and UPP-C [21, 36], and furthermore referred to as V17-C, A60-C, A79-C, A117-C, allA-C, MV1-C and MV2-C. In the first preliminary microscale cultivation (Supplementary File 1: Figure S 3) all tested P_DH_ variants except allA (represented by allA-C) presented a similar trend as observed for eGFP (Figs. 1 and 2), but with lower overall enhanced gene expression. MV1-C and MV2-C exhibited about 50% and 35% increased lipolytic activity, respectively, compared to the parental PDH-C, which is substantially lower than the 100% increase observed with eGFP. Unexpectedly, allA-C showed lower lipolytic activity than the parental PDH-C and all other strains with P_DH_ variants controlling *Ca*lB expression. This was also observed when cultivating the same strains in shake flasks under de-repressed conditions (Fig. 4).

Interestingly, in shake flask cultivations, all single- and multi-variants exceeded PDH-C activity levels during the first 20 h of de-repression (44 h sampling point), in the range of two to five-fold. Besides, MV1-C and MV2-C already presented a high production during the batch phase, being about 200% of the PDH-C and similar to the benchmark GAP-C. At the end of the cultivation, MV1-C and MV2-C showed 60% and 90% increased lipolytic activity compared to the parental PDH-C, respectively, which means 97 ± 10 and 111 ± 2 mg L^−1^ in terms of total protein. This distinct difference to the microscale cultivations (MV1-C 50% and MV2-C 35% increased) is presumably due to the more severe carbon-limiting conditions applied in shake flasks (*via* FeedBeads^®^ [36]) as compared to microscale, which are expected to result in a higher degree of promoter activation.

To conclude, for both secreted reporter proteins, two of the three evaluated P_DH_ multi-variants showed enhanced expression similar to intracellular eGFP. Nevertheless, it is known that specific results may depend on the specific expressed target protein [70]. Generally, for *Ca*lB production in shake flasks, multi-variants MV1-C and MV2-C show the most favorable expression profile, similar to those observed for eGFP. Astonishingly, with the lipase reporter, all variants show a seemingly less stringent carbon source repression, as a marked increase in reporter protein was observed for all tested variants after de-repression (44 h), which cannot be detected anymore after further cultivation under carbon-limiting conditions (58 h sampling point). At this stage, we cannot say if this is due to the general stress conditions caused by secretion of this lipase or other effects such as process conditions or intrinsic properties of the synthetic expression cassette such as mRNA stability.

### New multivariant MV2-C exhibited reliable high performance in fed-batch cultivation

In industrial-scale operations, the most used mode for RPP is still fed-batch cultivation. When a new bioprocess is developed, the workflow usually starts with strain engineering and small-scale screenings to identify preferred clonal variants, and ends with bioreactor cultivations, preferably in fed-batch mode [70, 71]. However, results from small-scale (microscale and shake flask cultivations) are not always transferable to the controlled environment that bioreactors provide. Therefore, according to the results detailed in the previous section, the best P_DH_ variant for producing *Ca*lB with the corresponding strain MV2-C, was selected to test its performance in a scalable cultivation strategy performed in fed-batch mode.

**Fig. 5:**
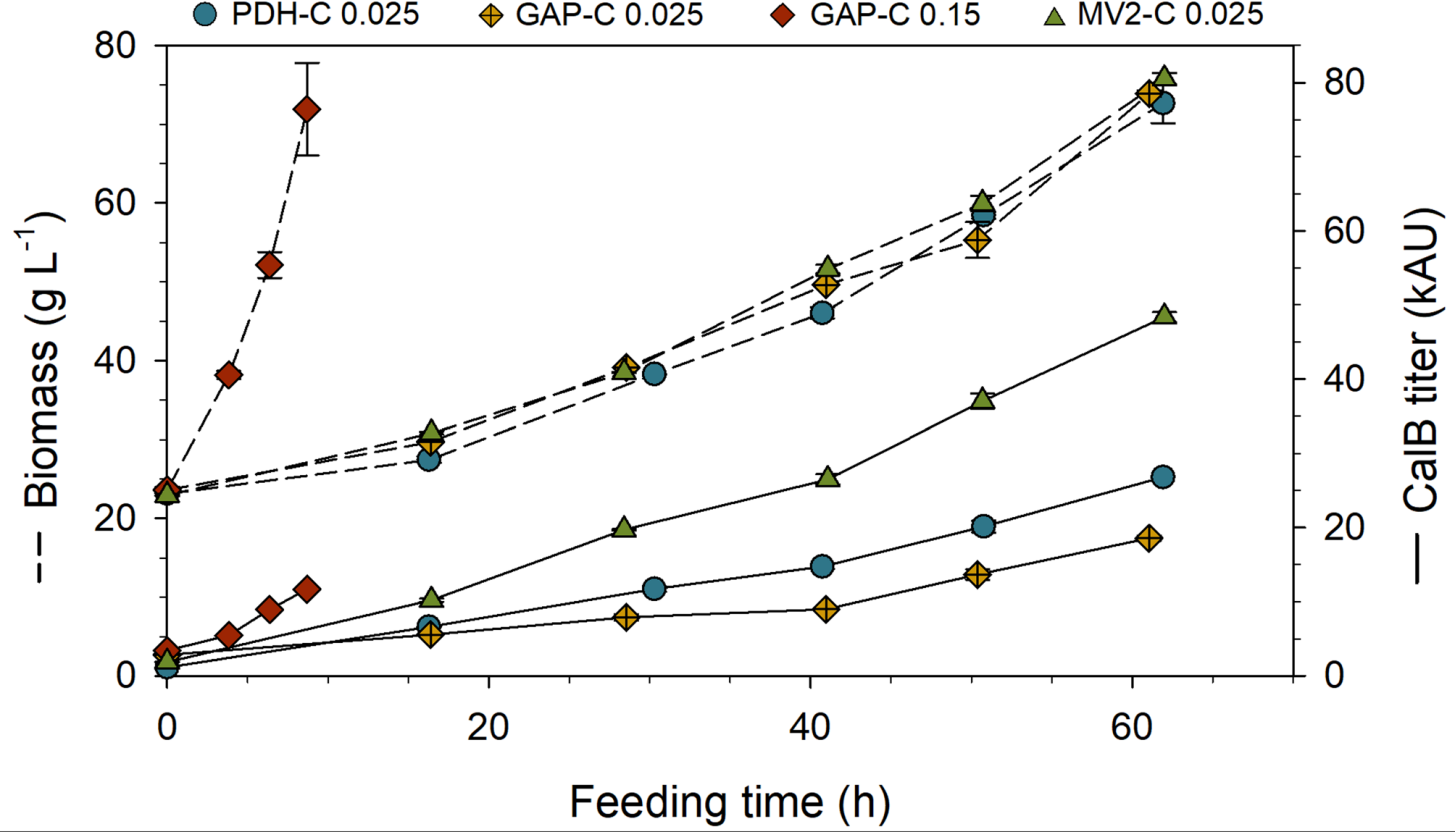
Validation of MV2-C in fed-batch cultivation under carbon-limiting conditions. Profiles of biomass concentration (DCW) and *Ca*lB production (titer) over glycerol feeding time at low *µ* (0.025 h^-1^) are shown for MV2-C together with the recently published results of GAP-C at high *µ* (0.15 h^-1^) and GAP-C and PDH-C at low *µ* (0.025 h^-1^) [36]. The error bars represent the standard deviation of analytical measurements

To compare MV2-C *Ca*lB production with its parental PDH-C and the benchmark GAP-C, a carbon-limiting fed-batch cultivation setting the specific growth rate (*µ*) at 0.025 h^−1^ was conducted. For all cultivations the target final biomass (80 g L^−1^ of dry cell weight (DCW) was successfully achieved (Fig. 5).

During the whole feeding time, MV2-C continuously produced *Ca*lB, which surpassed the production of all other cultivations, finalizing with a target protein titer 80% higher than PDH-C, and about three times higher compared to the best tested conditions with the P_*GAP*_. In terms of physiological parameters (Table 1), no differences could be detected between GAP-C, PDH-C and MV2-C at the same cultivation conditions, indicating that the higher *Ca*lB production by MV2-C did not negatively affect the cell fitness.

**Table 1:** Key process parameters of MV2-C fed-batch cultivation at low *µ* (0.025 h^-1^) compared to the recently published cultivation results of GAP-C high *µ* (0.15 h^-1^) and GAP-C and PDH-C low *µ* (0.025 h^-1^) [34]

	GAP-C 0.025	GAP-C 0.15	PDH-C 0.025	MV2-C 0.025
Real µ (h^−1^)	0.025	0.151	0.025	0.025
q_*s*_ (g_S_ g_x_^**−1**^ h ^−1^)	0.046	0.25	0.044	0.045
Y_x/s_ (g_x_^**−1**^ g_S_^**−1**^**)**	0.54	0.61	0.58	0.56
Titer (kU)	18.6	11.7	26.8	48.4
Y_p/x_ (U g_x_^**−1**^**)**	100.4	61.3	185.2	304.2
Specific production rate (q_p_, U g_x_^**−1**^ h^−1^)	2.5	9.4	4.6	7.7
Volumetric productivity (Q_v_, U L^−1^ h^−1^)	92.7	361.4	151.7	266.1

In particular, MV2-C presented a 1.7 and 3.1-fold increase in the specific production rate (*q*_*p*_) compared to PDH-C and GAP-C in fed-batch cultivations at low *µ* (0.025 h^−1^), respectively. Unsurprisingly, conducting the process under P_*GAP*_-optimized conditions (short process time at high *µ*) the GAP-C achieved a 20% higher *q*_*p*_ value even though the final enzyme titer was 4-fold lower. A similarly high increase, about 35%, can be also observed for volumetric productivity (*Q*_*V*_), since both parameters are time related. However, the product to biomass yield (Y_P/X_) of MV2-C was 60% and 500% times higher than for PDH-C and GAP-C at optimal conditions, respectively. It is substantial to mention, that P_*GAP*_-based production is growth-coupled, meaning that a high growth rate of biomass is required to obtain high levels of recombinant protein, which leads to a predetermined end of the bioprocess when the maximum cell density is achieved (< 10 h feeding time in the presented process). Additionally, the higher growth rate applied for the GAP-C-optimized conditions (*µ* 0.15 h^−1^) makes the process itself more demanding in terms of cooling and O_2_ supply, which increments the operational costs. In contrast, with the non-growth-coupled P_DH_-based system, time-related productivities might be lower, but working at slower µ allows for longer overall process times while still keeping up high production levels, resulting in a higher final protein titer, yield, and carbon efficiency.

The parental P_DH_ is a non-modified sequence fragment of the *K. phaffii HSP12* promoter region, and the general potential of this new promoter for methanol-independent processes was published previously [36]. To better understand the performance and potential of the *K. phaffii HSP12* promoter relative to the commonly used promoters in this yeast cell factory, additional insights can be found in a patent by Tan et al. (WO2020106599A1) [72]. Although not peer-reviewed, it describes a direct comparison between methanol-free processes using the *HSP12* promoter and methanol-induced processes driven by the *AOX1* promoter. The comparison also includes variations in cultivation time and signal peptides and showcases that the performance of the *HSP12* promoter (SeqID 1) is comparable to the methanol-induced *AOX1* promoter (SeqID 8).

Furthermore, the new engineered promoter variants presented in this study outperformed their parental promoter. In addition, the use of P_DH_-MV2 in a methanol-free fed-batch cultivation also presents a clear operational advantage over methanol-based bioprocesses regulated by P_*AOX1*_, which need additional steps for methanol adaptation and can present up to twice the oxygen consumption rate compared to glucose- or glycerol-based cultivations [73]. In terms of product yields, MV2-C achieved 0.460 ± 0.071 g L^−1^ of total protein in the broth with a low targeted biomass of 80 g L^−1^ (5.75 mg protein g^−1^ biomass). For comparison, a previously published study with a *Ca*lB-producing strain under P_*AOX1*_ control employing a methanol-induced process with a biomass concentration of 137 g L^−1^ (DCW) yielded 0.693 g L^−1^ [74]. A high product-to-biomass ratio is preferred for downstream processing and supports efforts to reduce waste from bioprocesses. When calculating the specific production, MV2-C produced 20% more total protein than the P_*AOX1*_-based bioprocess, an improvement that increases to 60% when considering the duration of the production phase. Therefore, P_DH_-MV2 can be positioned as a strong promoter and even rival P_*AOX1*_. However, although such comparisons provide some rough information about the specific performance of new systems compared to state-of-the-art tools, conclusions should be made cautiously due to significant differences in the cultivation strategies, which may not be optimal for either promoter, and also specific production strains of different studies cannot be directly compared due to differences in their respective genomes.

Overall, the promising results achieved with MV2-C in shake flasks could be validated in benchtop bioreactor scale following a low exponential growth rate strategy and obtaining highly similar results in terms of production rate increases. Considering that the applied fed-batch process was carbon-limited but not completely restrictive, the process might further be optimized by applying more severe carbon-limiting conditions, like the pseudo-starving strategy described by Bernat-Camps et al. [36]. This underlines the potential of the new promoter variants, such as the P_DH_ variant MV2-C. They are promising candidates for a non-growth-coupled process in which a very high titer and yield can be obtained in long bioprocesses with slow biomass growth, lower energy input, and high carbon efficiencies.

## Conclusions

In this work, different new strategic promoter engineering approaches were tested to broaden the toolbox of regulatory sequences available for RPP with *K. phaffii* not coupled to cell growth or depending on external inducers. Interestingly, as supported by previous publications, transcriptome analyses, and previous studies with *S. cerevisiae*, the *K. phaffii HSP12* promoter showed to be a good candidate for efficient methanol-independent gene expression. Therefore, the recently described *K. phaffii* promoter P_DH_ (352 bp upstream of the *HSP12* gene) was chosen as target for engineering to enhance pre-existing regulatory features and study sequence-function relationships of the sequence. With a semi-rational approach, first-generation variants with up to 50% higher expression than the parental promoter were successfully created and identified, and putative positive cis-acting elements could be correlated with the observed expression results. However, only some of them are in full agreement with the known *S. cerevisiae HSP12* regulatory circuit with e.g., multiple STRE binding sites. However, as the *ScHSP12* regulon was previously revealed to be a complex system with a multitude of promiscuous cis- and trans-acting elements, a holistic prediction of the P_DH_ regulatory network needs further data. Furthermore, even though alternative promoter lengths with 5’ extensions of the sequence had not shown higher eGFP expression in the initial evaluation [38], the lack of major negative cis-acting elements in the P_DH_ could indicate the presence of additional regulatory elements farther upstream of the *KpHSP12* gene.

The feasibility of combining beneficial mutations was demonstrated successfully, as second-generation variants were created, showing up to 2.5-fold higher intracellular eGFP production than the parental P_DH_. Characterization with a secreted reporter system (unspecific peroxygenase, *Abr*UPO) revealed an improvement of 60% compared to the parental promoter. Furthermore, the best variants were used to express and secrete the industrially relevant lipase B of *Candida antarctica* (*Ca*lB), resulting in up to 100% higher extracellular protein titer compared to the parental P_DH_ in shake flask cultivations. The best multi-variant *Ca*lB producer, MV2-C, was validated in a benchtop bioreactor fed-batch process, achieving production levels two-times higher than the parental PDH-C at the same carbon-limiting conditions.

Overall, these results highlight the benefits of large-scale promoter engineering approaches for investigating sequence–function relationships, which can consequently be used as a base to enhance promoter strength. The applied methodology introducing the positively acting 10 bp V-sequence block, further broadens the scope of efficient tools for engineering strong promoters for *K. phaffii*. Furthermore, new and efficient non-growth-coupled promoter variants have been generated and identified, and a first proof-of-concept for benchtop bioreactor scale application has been demonstrated. These promoters are not only valuable for the optimization of single gene expression and to fine-tune the expression of multiple genes but can also be applied in regulatory circuits in combination with constitutive promoters. Thus, creating complementary expression systems securing protein production over a broad period of time without the need for external inducers. Although, their full potential remains to be validated through promoter-specific process optimization for specific application examples in bioreactor scale operations, the performed tests presented in this work demonstrated promising transferability for different target proteins.

## Materials and methods

### Microbial strains, chemicals and media

Cloning work and plasmid propagation was done using the *E. coli* K12 strain TOP10F´. For initial evaluation of promoter variants using eGFP and the secretory production of *Abr*UPO-II (OJJ73116.1), the killer plasmid-free *K. phaffii* strain BSY10d*KU70* (*Mut*^*+*^) (bisy GmbH, Austria) was used as a host, which facilitated the comparison of regulatory elements by promoting targeted genomic integration due to reduced non-homologous end-joining activity [52]. Expression constructs for the secretory production of *Ca*lB were introduced into the plasmid-free *K. phaffii* strain BSYBG11 (*aox1-/Mut*^*S*^) (a deposit of the BioGrammatics BG11 strain at the bisy GmbH, Austria). All expression strains created during this study, with the respective base strains as well as target protein, promoter controlling expression and evaluated cultivation scale can be found in Supplementary File 1: Table S 1.

Unless stated otherwise, buffer, media components and chemicals were purchased either from Carl Roth GmbH (Karlsruhe, Germany), Fluka Chemia AG (Basel, Switzerland) or Merck GmbH (Darmstadt, Germany). Zeocin was acquired from InvivoGen-Eubio (Vienna, Austria) and used at a final concentration of 25 µg mL^−1^ for *E. coli* and 100 µg/mL_-1_ for *K. phaffii*.

Yeast minimal media for deep-well plate (DWP) cultivation was adapted from Weis et al. (2004) using buffered minimal media with glycerol as sole carbon source (abbreviated BMG, 200 mM potassium phosphate buffer pH 6, 13.4 g L^−1^ yeast nitrogen base and 0.4 mg/L biotin), at 1% (w/v) or 0.25% (w/v) in the batch and feeding phase, respectively [75]. Solid buffered minimal media supplemented with 2% (w/v) methanol (abbreviated BMM) was used for growth counter selection.

### Vectors and cloning work

All cloning work was done by in vitro recombination cloning, using the Gibson Assembly HiFi 1-Step Kit purchased from SGI-DNA Inc., now Telesis Bio Inc. (CA, US). Primers were used for amplification of DNA parts and to introduce 5´ and 3´ sequence homologies to parts adjacent to each other for isothermal assembly. Primers were designed using SnapGene (GSL Biotech LLC, Chicago, USA) to a Tm of 60 °C and ordered from IDT (Integrated DNA Technologies, Inc. Coralville, IA, USA).

The vector backbone for initial evaluation of promoter strength, eGFP_scan_entry, was assembled using parts of a bisy standard vector (pUC ori, Zeocin resistance cassette, stuffer sequence as placeholder for promoter), the *AOX*1 promoter (P_*AOX1*_) without core, the *AOX*1 terminator (*AOX1TT*) and an enhanced green fluorescent protein sequence (cycle-3-GFP, eGFP). The P_*AOX1*_ and *AOX1TT* are separated by a *Sm*iI restriction site, to facilitate targeted genomic integration of the linearized plasmid in the *AOX*1 locus by double cross over (replacement of the *AOX1* gene). The stuffer sequence upstream of the eGFP is flanked by two *Sap*I restriction sites for subsequent integration of promoter sequences (controls and P_DH_ variants). Promoter sequences used as controls (P_*GAP*,_, P_DH_) were amplified from bisy standard vectors and P_DH_ variants were ordered as synthetic DNA at Twist Bioscience (San Francisco, CA, USA) and amplified by PCR, prior to integration into the *Sap*I linearized eGFP_scan_entry. Constructs for the secretory production of *Abr*UPO (OJJ73116.1) were derived from the bisy proprietary standard vector pBSY6S1Z employing the P_DH_ to control transcription of the gene of interest (GOI) and a deletion variant of *S. cerevisiae* pro-pre α-mating factor (named S1) as signal peptide to enable secretion.

Constructs for the secretion of *Ca*lB were derived from the previously described pPpT4_Alplha_S-based expression vector used for the characterization of the parental P_DH_ [36]. P_DH_ variants were amplified by PCR from the P_DH_ -eGFP expression library and integrated into the PCR-amplified vector backbone in place of the parental P_DH_ to control expression of the GOI.

Prior to linearization and introduction into *K. phaffii* all plasmids were sequence verified by Sanger sequencing.

### Transformation, screening and rescreening procedures

Preparation and transformation of electrocompetent *K. phaffii* cells was done following the condensed protocol described by Cereghino et al. (2005) [76]. Electrocompetent cells were transformed with low amounts of of *Smi*I-linearized, purified DNA (1 µg) to avoid multicopy integrations. After regeneration in a 1:1 mixture of YPD and 1 M Sorbitol (at 28 °C, for 3 h) transformants were selected on YPD plates supplemented with Zeocin for 48–72 h.

For the initial evaluation of promoter strength using eGFP and *Abr*UPO, we facilitated targeted single-copy integration of the cassette into the *AOX*1 locus via double crossover (replacement of the *AOX1* gene and creating a Mut^S^ phenotype) by using a *Ku70* deficient strain. Therefore, only a small number of transformants per construct were picked for cultivation in BMG1% and verification of integration by BMM-counterselection. The cultures were grown to saturation for approximately 48 h before the feeding phase was started with the addition of 250 µL BMG0.5%. Cultures were incubated for further 48 h, with the addition of 50 µL BMG2.5% after 72 h of cultivation. For eGFP expression, based on the results of the initial screening, one transformant per construct from the linear range of the landscape showing a Mut^S^ phenotype was streaked for single colonies and uniform expression was confirmed by rescreening in biological triplicates inoculated from single colonies. Since precise detection of intracellular proteins is sensitive to cell culture density, the rescreening procedure of strains expressing eGFP was started with DWP pre-cultures to obtain uniform cell densities for all strains and wells. Pre-cultures were inoculated with single colonies and grown in BMG1% for approximately 48 h before 10 µL were used to inoculate the 250 µL BMG1% main culture. For intracellular eGFP production samples were taken after 48 h, 72 h and the end of the cultivation (96 h). For *Abr*UPO production, at least three different averagely performing clones from the initial landscape were subjected to a re-screening procedure in DWPs. Re-screening of strains secreting *Abr*UPO in DWPs was started with cell material of single colonies (without pre-cultures) and one sample was taken at end of the cultivation (96 h) for protein analysis. For all cultivations representative control strains having P_DH_ and P_*GAP*_ controlling model protein expression were included.

For the selection of *Ca*lB production strains, multiple transformants per construct (21 to 28 clones) were picked for cultivation in BMG1% for 48 h in DWP. Afterward, cultures were harvested, and the supernatant was analyzed for enzymatic activity. Single colonies of one representative transformant from the linear range of the landscape were used to confirm uniform expression by rescreening in biological triplicates. Cultivation of the rescreening was done as described for the eGFP transformants in a BMG1% batch followed by a feeding phase at 0.25% (w/v) glycerol. After verification of the activity the representative clone was used for shake flask and eventually fed-batch cultivations. As controls the previously described isogenic strains producing the *Ca*lB were used: GAP-C, UPP-C, and PDH-C [36].

### Computational in silico promoter analysis

The P_DH_ sequence (352 bp region upstream of the *K. phaffii HSP12* gene) was used to search for putative TFBSs using various web tools and open-access databases. Initial analysis of the promoter for putative TFBS was done using PROMO Version 3.0.2, narrowing down the search to factors and sites of fungi [77, 78]. To get a broader range of putative motifs, parameters were also extended to include factors and sites of all eukaryotes. Similarly, using JASPAR 2022, the sequence was scanned for all putative *S. cerevisiae* TFBS [79]. YEASTRACT: *Saccharomyces cerevisiae* was used for promoter analysis by cross-species comparison between the TFBS for *S. cerevisiae HSP12* and *K. phaffii* GS115 *HSP12*. The consensus binding motif of the carbon source-responsive zinc-finger transcription factor *Kp*Mxr1p (CAY71743.1, *Sc*Adr1p homolog) was taken from N.C. YEASTRACT: *Komagataella phaffii* [80, 81].

### Shake flask cultivation

The shake flask cultivation protocol was adapted from Bernat-Camps et al. [36]. Briefly, shake flasks containing 250 mL of BMG1% were inoculated from an overnight culture in YPG to an initial OD of 0.2. After 24 h of cultivation at 25 °C and 180 rpm, total glycerol consumption was confirmed by HPLC, and a single glycerol FeedBead^®^ (SMFB12001, Kuhner Shaker, Basel, Switzerland) was added to each shake flask. The cultivations were run per triplicate for 72 h in total, taking four samples over the cultivation time to check OD and enzyme activity in the supernatant.

### Protein analysis: fluorescence measurements and enzyme assays

For fluorescence measurements, DWP cultivations cultures were diluted 1:20 with ddH_2_O in a total volume of 200 µL in 96-well microplates (Nunc MicroWell 96-well optical-bottom plates with a polymer base, black; Thermo Fisher Scientific). Optical density (OD_600_) and fluorescence of eGFP was determined at 600 nm and 488 nm excitation – 507 nm emission, respectively, in a CLARIOstar Plus plate reader (BMG Labtech, Ortenberg, Germany). Fluorescence units (RFU) were corrected by OD_600_ to account for differences in culture growth. Relative promoter activity of variants and controls was calculated as OD_600_ corrected fluorescence units normalized to the respective value of the parental P_DH_ (RFU OD_600_ ^−1^ P_DH_^−1^) for each timepoint. The amount of secreted *Abr*UPO was determined by measuring the oxidation of ABTS in the supernatant of DWPs and shake flasks cultivations in microtiter format, similar to previously described procedures [82]. Briefly, the reaction was done in sodium citrate buffer (200 mM, pH 4.5) using 1.16 mM ABTS and 0.26 mM H_2_O_2_. The change in absorbance at 405 nm was followed for 15 min, and the extinction coefficient of oxidized ABTS (36,000 M^−1^ cm^−1^) was used to calculate volumetric activity. The amount of secreted *Ca*lB was determined by measuring the hydrolysis of p-nitrophenyl-butyrate (pNPB, Merck, Darmstadt, Germany) in a reaction containing Tris-HCl buffer (300 mM, pH 7.4), 4.83 mM p-NPB and 0.93% acetone, as described previously [36]. The change in absorbance at 405 nm was followed for two minutes and the extinction coefficient of pNPB (9.594 mM^−1^ cm^−1^) was used to calculate volumetric activity. One unit was defined as the amount of enzyme needed to release 1 µmol p-nitrophenol min^−1^.

For *Abr*UPO and *Cal*B, total protein concentration was determined using Bradford assay with BSA as standard.

### Fed-batch cultivations

MV2-C fed-batch cultivation was carried out in a 5 L *Biostat B* bioreactor (Sartorius Stedim, Goettingen, Germany) with an initial volume of 2 L. The working conditions were set and controlled all over the cultivation at: temperature 25 °C, pH maintained at 5 by 15% (v/v) NH_4_OH addition, aeration 2 L min^−1^ and dissolved oxygen (DO) was controlled over 30% by agitation rate cascade. Media recipes for batch and fed-batch media can be found elsewhere [83]. Deviating, the carbon source in fed-batch phase was a 400 g L^−1^ glycerol solution combined with key micro and macronutrients. The cultivation process and medium compositions were previously described by Bernat-Camps et al. [36] where a carbon-limiting fed-batch at a constant specific growth rate (*µ*) of 0.025 h^−1^ was initiated upon glycerol depletion in the batch phase. The mass balance calculations for the pre-programmed exponential feeding rate can be found elsewhere [84, 85]. The fed-batch cultivation was stopped when the goal biomass of 80 g L^−1^ of DCW was reached. Several samples over the cultivation were taken to measure biomass growth, protein production and metabolites or substrate accumulation. Off-gas from the bioreactor was analyzed with a *BlueInOne FERM* gas analyzer (BlueSens, Herten, Germany), whose measurements of O_2_ and CO_2_ molar fraction and absolute humidity were used to calculate key respirometric parameters.

### Biomass determination

The biomass growth in micro- and small-scale cultivations was determined by measuring the optical density at 600 nm (OD_600_) in triplicates in a spectrophotometer. In fed-batch cultivation though, biomass growth was followed in terms of DCW, using the protocol described by Cos et al. (2005) [86]. The relative standard deviation (RSD) in all determinations was lower than 5%.

### Quantification of the carbon source and byproducts

Presence of remaining carbon source in the supernatant of shake flask cultivations and its accumulation besides metabolites production in fed-batch cultivation were determined by HPLC (Dionex Ultimate 3000, Dionex, Sunnyvale, CA, United States) with an ionic exchange column (ICSep ICE-COREGEL87 H3, Transgenomic Inc., Omaha, NE, United States) [87].

### Consistency test and data reconciliation

Mass balance and electron balance of fed-batch cultivation were calculated and evaluated according to Garcia-Ortega et al., (2016) obtaining less than 5% of deviation [29]. All calculated parameters were reconciliated applying the statistics calculations found elsewhere [88, 89, 89].

## Supplementary Information

Additional file 1.

## Data Availability

No datasets were generated or analysed during the current study.
