# Supplementary material for: Next-generation stress-inducible Komagataella phaffii promoter variants
Source: Microb Cell Fact. 2025 Oct 30;24:228. doi: 10.1186/s12934-025-02825-7 (PMC12573857; doi:10.1186/s12934-025-02825-7)
Supplement: Supplementary file 1 — Additional file 1. [file 12934_2025_2825_MOESM1_ESM.docx]

Electronic Supplementary Information for

**Next-generation stress-inducible *Komagataella phaffii* promoter variants**

Katharina Ebner^1†^, Núria Bernat-Camps^2,3†^, Simona Scheipel^1^, Corina Dörner^1^, Francisco Valero^2,3^, Anton Glieder*^4^, Xavier Garcia-Ortega^2,3^

*^1^Bisy GmbH, Wuenschendorf 292, 8200 Hofstaetten/Raab, Austria.*

*^2^Department of Chemical, Biological and Environmental Engineering, Universitat Autònoma de Barcelona, 08193 Bellaterra (Cerdanyola del Vallès), Spain.*

^3^ *Austrian Centre of Industrial Biotechnology (ACIB), Graz, Austria*

*^4^ Institute of Molecular Biotechnology, Graz University of Technology, NAWI Graz, Petersgasse 14, 8010 Graz, Austria*

*^†^ These authors contributed equally to this work.*

Corresponding author: Anton Glieder

Phone: +43 (316) 873 - 4074

e-mail: a.glieder@tugraz.at

**Keywords:** *Komagataella phaffii* (*Pichia pastoris)***,** promoter engineering, recombinant protein production, methanol-free expression system, stress response promoter, *HSP12* promoter, block-scanning technologies


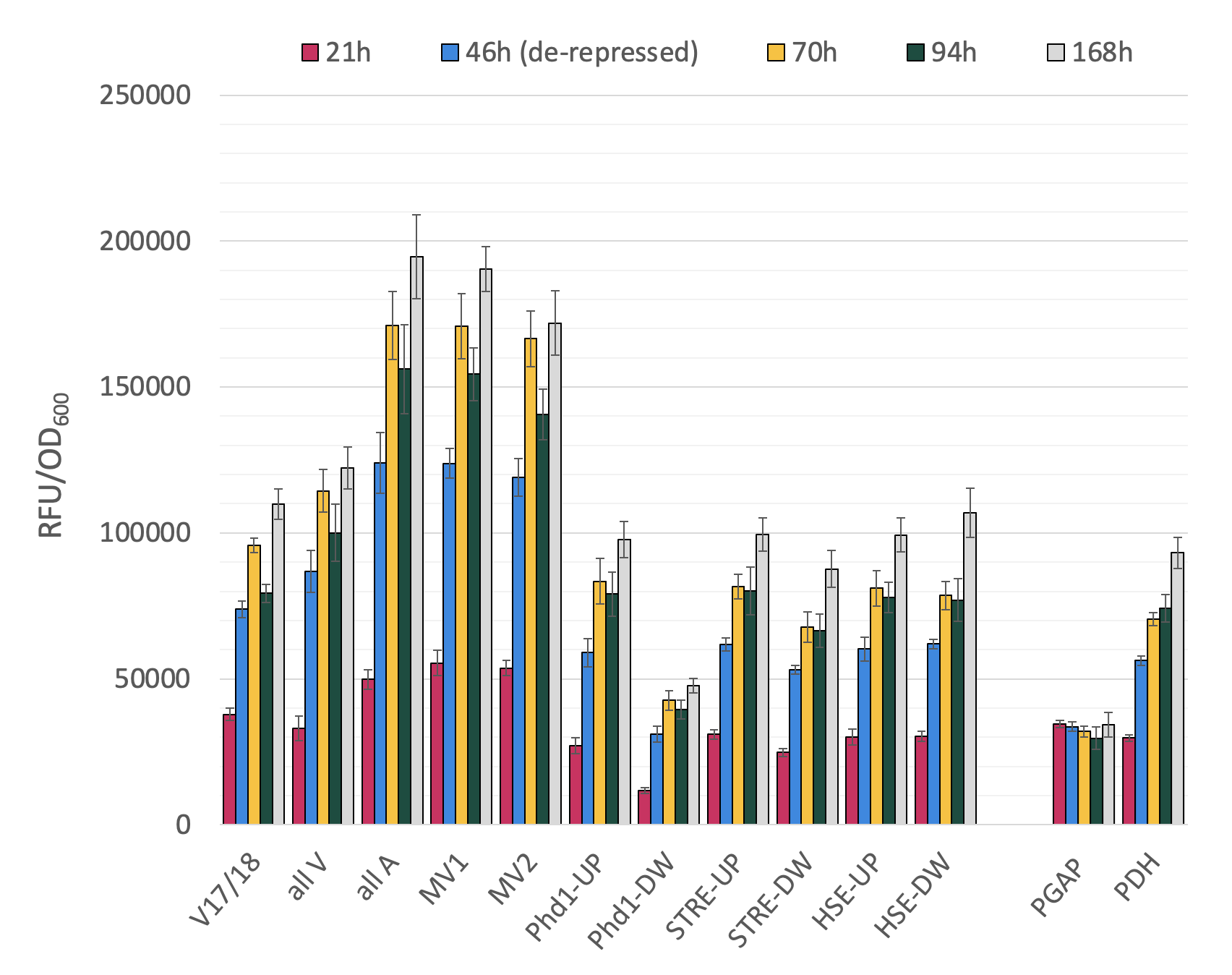


Figure S 1: Initial evaluation of second generation P_DH_ variants with intracellular eGFP in high-throughput microscale. Eleven second generation variants were created, five multicombination variants (combination of interesting V- and A-variants) and six variants with additional cis-acting elements as replacement of the wildtype sequence. The cis-acting elements HSE (`CCCCT´), STRE (`GAA´) and the Phd1 binding site motif (`TGCA´) were positioned in four consecutive repeats alternating between the two DNA strands at two different locations in the most upstream part of the PDH (U = 1-50 bp or D = 50-100 bp). OD_600_ corrected fluorescence is shown as measure of promoter activity and was determined at five different points of the cultivation, starting as soon as 21 h. and ending after 168 h. Given values represent the mean of biological triplicate cultivations of a representative strain in one deep-well plate, standard deviations are shown.


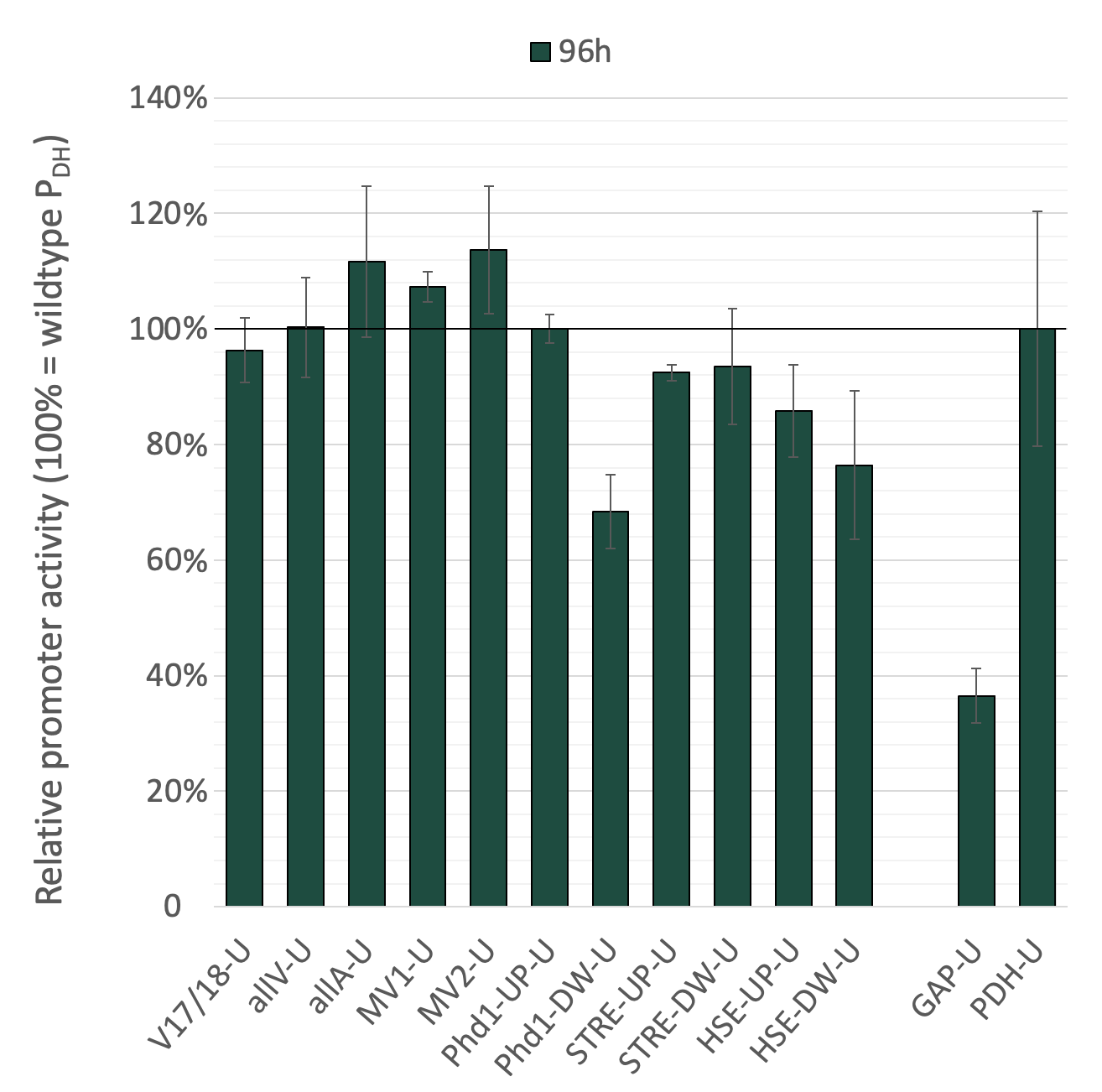


Figure S 2: Microscale rescreening of *Abr*UPO expression by second-generation P_DH_ variants. (A) Relative promoter activity is shown as mean enzymatic activity in the supernatant normalized to value of the parental PDH (ΔAbs*min^-1^*P_DH_^-1^). Values represent the mean of at least three different representative strains cultivated in biological triplicates, error bars indicate standard deviation. All values are corrected by the measurements for the control strain not expressing any target protein.


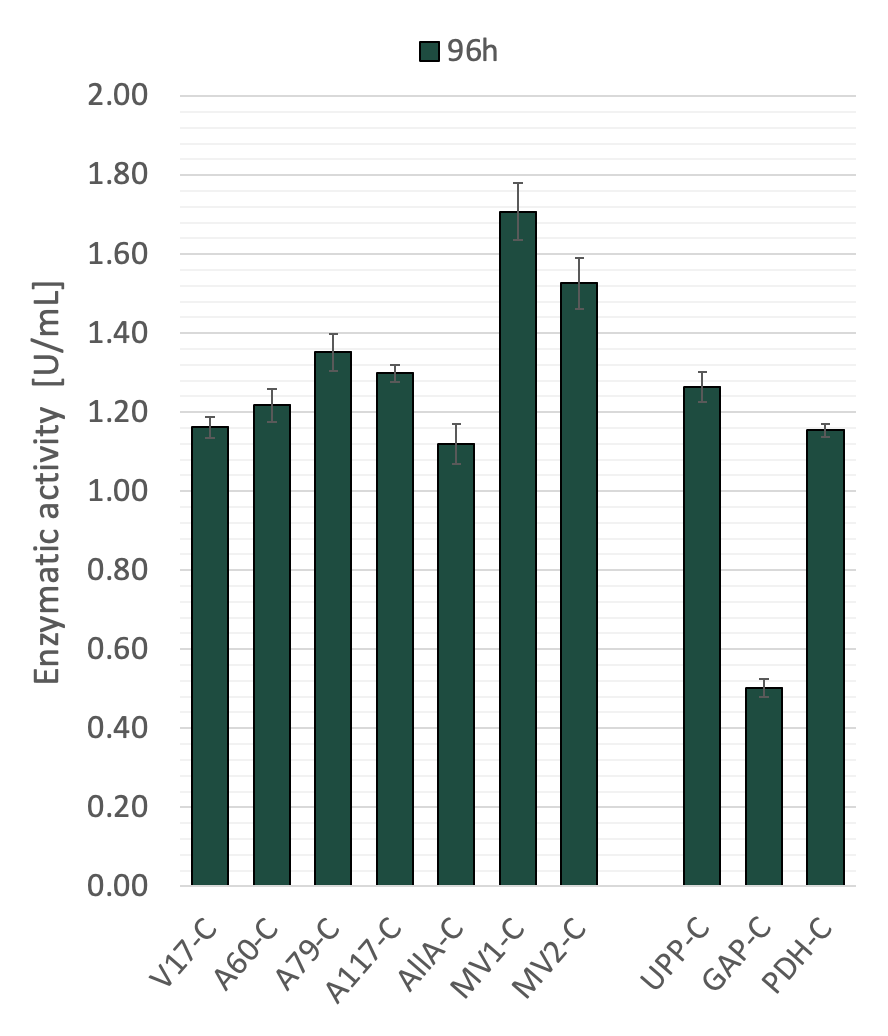


Figure S 3: Microscale rescreening of *Ca*lB expression by selected P_DH_ variants. Enzymatic activity in the supernatant is shown as the change in absorbance at 405 nm upon hydrolysis of p-nitrophenyl-butyrate. Values represent the mean of at least three different representative strains cultivated in biological triplicates, error bars indicate standard deviation. All values are corrected by the measurements for the control strain not expressing any target protein.

Table S 1: Overview of all expression strains created during this study.

| **Background strain** | **Classification** | **Promoter or sequence modifications** | **Reporting protein** | **Number of strains** | **Cultivation mode** | | | **Specific clone name** |
| --- | --- | --- | --- | --- | --- | --- | --- | --- |
|  |  |  |  |  | **DWP** | **Shake flask** | **FB** |  |
| **BSYBG10Δ*ku70*** (*AOX1* knock-out upon expression cassette integration) | V-library | 10 bp exchange | eGFP | 35 | X | - | - | - |
|  | A-library | 3A's exchange | eGFP | 117 | X | - | - | - |
|  | Multi-Variants | See Figure 2A | eGFP | 11 | X | - | - | - |
|  | Benchmarks | P*_GAP_* | eGFP | 1 | X | - | - | - |
|  |  | P_DH_ | eGFP | 1 | X | - | - | - |
|  | Multi-Variants | See Figure 2A | *Abr*UPO | 8 | X | - | - | - |
|  |  | A1, A35, A37, A60, A79, A117 | *Abr*UPO | 1 | X | X | - | AllA-U |
|  |  | A60, A79, A117 | *Abr*UPO | 1 | X | X | - | MV1-U |
|  |  | V17, V18, A79, A117 | *Abr*UPO | 1 | X | X | - | MV2-U |
|  | Benchmarks | P*_GAP_* | *Abr*UPO | 1 | X | X | - | GAP-U |
|  |  | P_DH_ | *Abr*UPO | 1 | X | X | - | PDH-U |
| **BSYBG11** | V-library | V17 | *Ca*lB | 1 | X | X | - | V17-C |
|  | A-library | A60 | *Ca*lB | 1 | X | X | - | A60-C |
|  |  | A79 | *Ca*lB | 1 | X | X | - | A79-C |
|  |  | A117 | *Ca*lB | 1 | X | X | - | A117-C |
|  | Multi-Variants | A1, A35, A37, A60, A79, A117 | *Ca*lB | 1 | X | X | - | AllA-C |
|  |  | A60, A79, A117 | *Ca*lB | 1 | X | X | - | MV1-C |
|  |  | V17, V18, A79, A117 | *Ca*lB | 1 | X | X | X | MV2-C |
|  | Benchmarks | P*_GAP_* | *Ca*lB | 1 | X | X | X | GAP-C |
|  |  | P_DH_ | *Ca*lB | 1 | X | X | X | PDH-C |

The base strain, expressed target protein, promoter controlling expression, number of created strains, evaluated cultivation scale and specific clone name (if specified) are given. DWP, deep well plate; FB, bioreactor operated in fed-batch mode.
